# Normal adult survival but reduced *Bemisia tabaci* oviposition rate on tomato lines carrying an introgression from *S. habrochaites*

**DOI:** 10.1186/s12863-014-0142-3

**Published:** 2014-12-24

**Authors:** Alejandro F Lucatti, Fien RG Meijer-Dekens, Roland Mumm, Richard GF Visser, Ben Vosman, Sjaak van Heusden

**Affiliations:** Wageningen UR Plant Breeding, Wageningen University and Research Centre, PO Box 386, 6700 AJ Wageningen, The Netherlands; Graduate School Experimental Plant Sciences, Wageningen Campus. Droevendaalsesteeg 1, 6708 PB Wageningen, The Netherlands; Plant Research International, Business Unit Bioscience, Wageningen University and Research Centre, P.O. Box 619, 6700 AP Wageningen, The Netherlands; Netherlands Metabolomics Centre, Einsteinweg 55, 2333 CC Leiden, The Netherlands; Bayer CropScience Vegetable Seeds, Napoleonsweg 152, 6083 AB Nunhem, The Netherlands

**Keywords:** Whitefly, Life history traits, Fine mapping, *Tv-1*, *Tv-2*, Trichome type IV

## Abstract

**Background:**

Host plant resistance has been proposed as one of the most promising approaches in whitefly management. Already in 1995 two quantitative trait loci (*Tv-1* and *Tv-2*) originating from *S. habrochaites* CGN1.1561 were identified that reduced the oviposition rate of the greenhouse whitefly (*Trialeurodes vaporariorum*). After this first study, several others identified QTLs affecting whitefly biology as well. Generally, the QTLs affecting oviposition were highly correlated with a reduction in whitefly survival and the presence of high densities of glandular trichomes type IV. The aim of our study was to further characterize *Tv-1* and *Tv-2*, and to determine their role in resistance against *Bemisia tabaci.*

**Results:**

We selected F_2_ plants homozygous for the *Tv-1* and *Tv-2* QTL regions and did three successive backcrosses without phenotypic selection. Twenty-three F_2_BC_3_ plants were phenotyped for whitefly resistance and differences were found in oviposition rate of *B. tabaci*. The F_2_BC_3_ plants with the lowest oviposition rate had an introgression on Chromosome 5 in common. Further F_2_BC_4,_ F_2_BC_4_S_1_ and F_2_BC_4_S_2_ families were developed, genotyped and phenotyped for adult survival, oviposition rate and trichome type and density. It was possible to confirm that an introgression on top of Chr. 5 (*OR-5*), between the markers rs-2009 and rs-7551, was responsible for reducing whitefly oviposition rate.

**Conclusion:**

We found a region of 3.06 Mbp at the top of Chr. 5 (*OR-5*) associated with a reduction in the oviposition rate of *B. tabaci*. This reduction was independent of the presence of the QTLs *Tv-1* and *Tv-2* as well as of the presence of trichomes type IV. The *OR-5* locus will provide new opportunities for resistance breeding against whiteflies, which is especially relevant in greenhouse cultivation.

**Electronic supplementary material:**

The online version of this article (doi:10.1186/s12863-014-0142-3) contains supplementary material, which is available to authorized users.

## Background

Tomato is one of the most important vegetables worldwide. It is host for a broad range of pathogens and pests. Among the pests affecting tomato production whiteflies are the most important in terms of costs and distribution. There are more than 1500 species of whiteflies [1], of which *Bemisia tabaci* Group Mediterranean-Middle East-Asia Minor I and *Trialeurodes vaporariorum* (Westwood) are the biggest threats in commercial tomato production. *Bemisia tabaci* affects tomato production directly (i.e. phloem consumption, irregular ripening of the fruits) and indirectly (virus transmission) causing yield losses that can range from 50% to 100% of the potential production [2,3].

Among the possible control methods, host plant resistance has been proposed as one of the most promising for insect pest management [4,5]. Resistance to whiteflies was found in several wild relatives of tomato (*Solanum pennellii*, *S. habrochaites, S. lycopersicum var. cerasiforme, S. pimpinellifolium, S. galapagense*) [6-14]. In these species, whitefly resistance is associated with the presence of high densities of glandular trichomes (type I, IV and VI) and with the presence of specific secondary metabolites (a.o. 7-epizingiberene, 2-tridecanone, and acyl sugars) [14-17]. The species *S. habrochaites* contains accessions (formerly known as *Lycopersicon hirsutum fr. glabratum*) that accumulate methyl ketones, of which the synthesis is located in the glandular head of type VIc trichomes [18-20]. *Solanum habrochaites* also contains accessions (formerly known as *Lycopersicon hirsutum fr. typicum*) that accumulate sesquiterpenes which are synthesised in type IV trichomes [17]. In *S. pennellii*, *S. pimpinellifolium* and *S. galapagense* the synthesis of acyl sugars is associated with the presence in high densities of trichomes type IV [12,15,16,21]. Although also some accessions of *S. cheesmaniae* accumulate high levels of acyl sugars, they lack type IV trichomes [14]. The *Mi1-2* gene, which confers resistance to several species of root-knot nematodes (*Meloidogyne* spp.) [22], plays a role in the resistance against insects, e.g. some isolates of potato aphid (*Macrosiphum euphorbiae* Thomas) [23,24], the sweet potato whitefly (*B. tabaci*) [25] and the tomato psyllid (*Bactericerca cockerelli*) [26]. This resistance is independent of the presence of glandular trichomes and acyl sugar concentration [27].

QTL mapping studies have been carried out to identify genomic regions involved in whitefly resistance. Maliepaard *et al.* [28] focused on resistance against the greenhouse whitefly *T. vaporariorum* (Westwood) from *S. habrochaites* (CGN1.1561) and identified two QTLs reducing whitefly oviposition rate (*Tv-1* on Chr. 1 and *Tv-2* on Chr. 12) together with two QTLs related to trichome type IV density (*TriIV-1* on Chr. 5 and *TriIV-2* on Chr. 9) and one QTL for trichome type VI density (*TriVI-1* on Chr. 1). After this first study, others have explored different resistance sources and more QTLs were identified. A summary of the QTLs related to whitefly resistance in tomato is given in Table 1. The use of backcross introgression lines (ILs) was also proposed as a method to identify genomic regions important for whitefly resistance. These ILs helped to identify regions and genes involved in traits related to insect resistance, like the production of monoterpenes, sesquiterpenes and acyl sugars [29-32]. However, they failed to identify regions associated to whitefly resistance in terms of adult survival or oviposition rate [33], supporting the observations from the QTL mapping studies that whitefly resistance is polygenic inherited and possibly epistatic interactions play a role as well. Except the QTLs described by Maliepaard *et al.* [28], all other QTLs affecting whitefly oviposition were highly correlated with a reduction in whitefly survival and/or to high densities of trichomes type IV, suggesting that the low oviposition rate is the consequence of a low survival rate [16,33,34]. To study resistance mechanisms affecting whitefly oviposition rate exclusively, we focused on the further characterization of the QTLs identified by Maliepaard *et al.* [28], and determined their role in resistance against *Bemisia tabaci.*

**Table 1:** **Overview of the QTLs found associated to whitefly resistance in tomato**

**Trait**	***QTL***	**Chr.**	***Resistance donor***	**% Explained**	**References**
Adult survival (*B. tabaci*)	*Wf-1*	2	*S. galapagense* (PRI95004)	54.1	[16]
	*Wf-2*	9		14.8	
	*Wf-I*	1	*S. pennellii* (LA3791)	12.1	[34]
	*Wf-III*	3		15.6	
	*Wf-IV*	4		12.3-30.7	
	*Wf-VI*	6		10.1	
Oviposition rate (*B. tabaci*)	*Wf-1*	2	*S. galapagense* (PRI95004)	41.7	[16]
	*Wf-2*	9		11.1	
	*R2/9*	9	*S. habrochaites* (LA1777)	55.2	[33]
	*R1/10*	10		15	
	*R3/11a*	11		52.9	
	*R4/11b*	11		43.3	
	*Wf-IV*	4	*S. pennellii* (LA3791)	10.3-29.6	[34]
	*Wf-VI*	6		13.9	
	*Wf-X*	10		10	
Oviposition rate (*T. vaporariorum*)	*Tv-1*	1	*S. habrochaites* (CGN1.1561)	6.4	[28]
	*Tv-2*	12		8	
Pre-adult survival (*B. tabaci*)	*Wf-1*	2	*S. galapagense* (PRI95004)	13.3	[16]
Density of trichome type IV	*Wf-1*	2	*S. galapagense* (PRI95004)	66.3	[16]
	*Wf-2*	9		8.7	
	*TriIV-1*	5	*S. habrochaites* (CGN1.1561)	n.d.	[28]
	*TriIV-2*	9		n.d.	
	*R2/9*	9	*S. habrochaites* (LA1777)	69.7	[33]
	*R1/10*	10		22.5	
	*R3/11a*	11		69	
	*R4/11b*	11		n.d.	
	*TA2A*	2	*S. pennellii* (LA0716)	2.6	[35]
	*3A*	3		5.1	
	*TA4*	4		5.2	
	*6A*	6		4.7	
	*7B*	7		2.8	
	*10A*	10		4.6	
	*11A*	11		8.1	
Density of trichome type VI	*TriVI-1*	1	*S. habrochaites* (CGN1.1561)	n.d.	[28]

% Explained = percentage of variance explained by the QTL.

## Methods

### Plant materials and growing conditions

**Figure 1:**
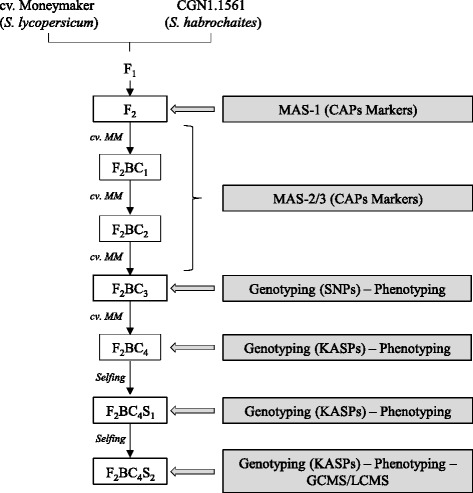
**Overview of the pedigree scheme and plant material development.**

The study was based on the F_2_ offspring population that was created by Maliepaard *et al*. [28], it was obtained by self-pollination of a single F_1_ plant that was derived from a cross between *S. lycopersicum* (cv. Moneymaker) and *S. habrochaites* (CGN1.1561). We have sown again individuals of this F_2_ population and selected plants that were homozygous for either one or both QTLs associated to a reduction in oviposition rate using Cleaved Amplified Polymorphisms (CAPs) markers (Table 2). The selected F_2_, BC_1_ and BC_2_ plants were backcrossed with *S. lycopersicum* (cv. Moneymaker) for three generations. Plants were chosen containing at least one of the markers flanking the QTLs. The obtained F_2_BC_3_ and F_2_BC_4_ families were genotyped and phenotyped for adult survival and oviposition rate. Selected F_2_BC_4_ plants were selfed to obtain F_2_BC_4_S_1_ plants and F_2_BC_4_S_2_, which were also genotyped and phenotyped. An overview of the material development is shown in Figure 1.

**Table 2:** **Primers for CAPs analysis**

**Marker name**	**Chr.**	**Primer sequence**	**Restriction enzyme**
TG59	1	AACTCTACGCTGCACTGCTG	*Hpa* II
		CTGAAGCTCCACCTTGAGGTG	
TG17	1	GGTCTTCCCTTCGTCATTCAT	*Hpy*CH4 IV
		GTTATTCGGTTCTTGTTCTTCACG	
CD2	12	CAGCTGCAACTCCACTACCA	*Mwo* I
		GGGCTTGAAGAACTGCACTC	
TG68	12	TTTGATTACACCTGCCTTTACATA	*Dde* I
		CATGTCAAGGGGATTGAACA

The tomato plants were grown in a greenhouse in Wageningen, The Netherlands (20 ± 2°C, 70% RH, 16/8 h day/night) in 14 cm diameter pots filled with soil compost. The plants were fertilized twice a week with standard fertilizer for tomato and watered once a day. When the plants were five weeks old, they were transferred to an insect proof greenhouse. The greenhouse temperature was increased slowly from 20 to 27°C to allow plants to adapt to the higher temperature (27 ± 2°C, 70% RH, 16/8 h day/night) used during the infestation that took place one week after transfer.

### Insect rearing

A non-viruliferouswhitefly rearing (*Bemisia tabaci* Group Mediterranean-Middle East-Asia Minor I) was maintained on the susceptible tomato cultivar Moneymaker at Wageningen UR Plant Breeding, Wageningen, The Netherlands. The initial inoculum was obtained from a rearing at the Laboratory of Entomology, Wageningen UR, Wageningen, The Netherlands.

### No-choice experiment

Whiteflies (four days old) were anesthetized using CO_2_. Five females were selected under a binocular and put in a clip-on cage (2.5 cm diameter and 1.0 cm high). Three cages per plant were attached to the first to third fully expanded leaf counting from the top. Five days after inoculation, the number of living and dead whiteflies was recorded and living whiteflies were removed. The number of eggs was counted, and the Oviposition rate (OR) and Adult survival (AS) were calculated according to Bas *et al.* [36]. In these calculations mortality is assumed constant over time [37]. For the analysis of AS in the F_2_BC_3_ population, a Kruskal-Wallis analysis of variance was used [38]. A square root transformation was applied to oviposition rate (OR) prior to the data analysis and analysed by one-way ANOVA followed by a least significant difference (LSD) test.

### DNA isolation and genotyping

Genomic DNA was extracted from young leaflets using the micro-prep DNA extraction protocol [39]. The DNA concentration was adjusted to 50 ng/ul. For molecular marker analysis, three types of marker assays were used: CAPs, a custom made Infinium bead array and KASPar (KBiosciences Competitive Alelle-Specific PCR).

For CAPs the PCR reactions were carried out in a final volume of 20 μl, containing 50 ng of genomic DNA, 0.04 μl of DreamTaq polymerase (Fermentas), 2 μl 10X DreamTaq buffer (Fermentas), 0.4 μl of dNTP (5 mM) and 1 μM of each primer (20 pmol). The cycling profile was: 94°C for 3 min, followed by 30 cycles at 94°C for 30 s, 55°C for 30 s, and 72°C for 1 min, and a final extension step at 72°C for 10 min. Aliquots (5 μl) of the amplified products were digested for at least one hour at 37°C in a final volume of 15 μl with 0.5 μl of the appropriate restriction enzyme, using the buffer recommended by the supplier. Amplification and digestion products were analysed by agarose gel electrophoresis (1.5% TBE, agarose) and visualized by GelRed® staining. In Table 2 the primer sequences and the restriction enzymes used are shown.

For genome wide SNP marker analysis, an Infinium bead array was used [40]. On this array, 5528 tomato SNPs were present. Marker analysis was carried out by Service XS Leiden, The Netherlands, according to the Illumina® Infinium HD Ultra Assay protocol (www.illumina.com). After removing missing data and monomorphic markers, 1166 SNP markers were used in the analysis. For fine mapping of the target regions, we developed KASPar assays based on SNP markers that were on the array. The chromosomal positions are according to International Tomato Annotation Group (ITAG) Release 2, official annotations on the SL2.31 version of the tomato genome [41] (www.solgenomics.net). The sequences flanking the SNPs can be found on http://www.plantbreeding.wur.nl/Publicaions/SNP/4072SNP-Sequences.xlsx [39]. The KASPar assays were run by the van Haeringen lab (VHL), Wageningen, the Netherlands.

### Trichome description

Trichomes present on the abaxial side of the leaf were classified according to type [42]. For an estimation of trichome density, the abaxial part of three leaflets was observed under a binocular microscope and a visual scale was used to describe it. The scale used was adapted from Simmons and Gurr [43] and consisted of four categories: 3, Abundant (>5 per mm2); 2, sparse (5–1 per mm2); 1, very sparse (<1 per mm2), and 0, absent.

### GC-MS analysis

The F_2_BC_4_S_2_ plant were analysed for the presence and concentration of methylketones. From each plant one complete leaf (second fully expanded leaf from the top of the plant) was cut, placed immediately into an aluminium envelope and frozen in liquid nitrogen. Each sample was ground to a freeze-dried powder and stored at −80°C until processing. Tree biological replicas were used for the analysis. Each replica consisted on the mix of 5 plants per recombinant class. Per replica, 400 mg of leaf powder was put into a reaction tube with 3 ml of anhydrous dichloromethane (>99.8%, Sigma-Aldrich) as solvent and 0.75 μg per ml heptadecanoic acid methyl ester was added as internal standard. The samples were homogenized using a vortex and centrifuged at 1500 rpm for 10 min. The supernatant was filtered through a soft glass column (Pasteur capillary pipette), which contained 1 cm of silanized glass wool fibres and 2 cm sodium sulphate (Na_2_SO_4_) powder as filter. Samples were injected using a 7683 series B injector (Agilent Technologies) into a 7890 A GC (Agilent Technologies) coupled to a 5975 C MSD (Agilent Technologies). Chromatography was performed using a Zb-5MS column (Phenomenex, 30 m, 0.25 mm inner diameter, and 0.25 μm film thickness) with 5 m retention gap. Injection temperature was 250°C, and temperature of column was programmed at 45°C for 1 min, increased by 10°C min^−1^ to 300°C, and kept at 300°C for 7 min. Column flow was set at 1 ml min^−1^, using Helium as carrier. The column effluent was ionised and mass spectra was obtained from 35–400 m/z. MetAlign metabolomics software package (www.metalign.nl) was used to perform peak alignment and noise reduction, and MSClust software package (www.metalign.nl) was used for data reduction by clustering several peaks into putative metabolites. Putative metabolites were identified corresponding the obtained mass spectra to the NIST library (National Institute of Standards and Technology, Gaithersburgh, MD, USA), the Wiley online library, and the Wageningen Natural compounds spectral library. Prior to statistical analysis, the metabolites were Log transformed and auto scaled to the mean. To select metabolite compounds putatively related to whitefly preference a t-test, followed by False Discovery Rate correction [44].

### Statistical analysis

All statistical procedures were performed using the statistical software package GenStat 16th edition. A T-test followed by a False discovery Rate [44] was done per marker to define the region associated to the reduced oviposition rate.

## Results

**Figure 2:**
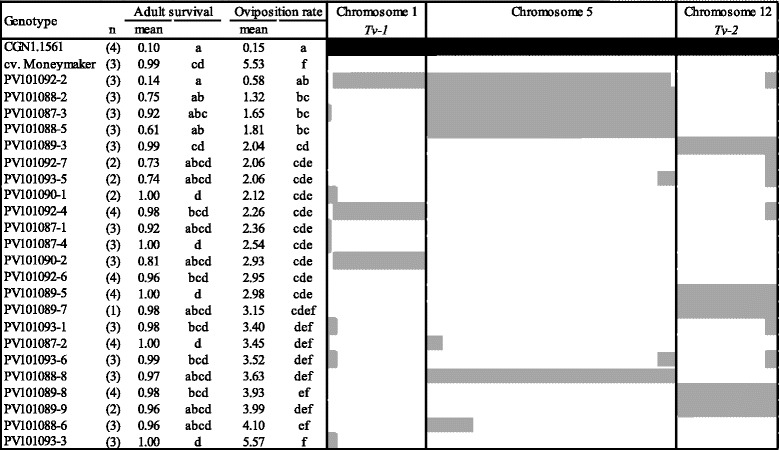
**Whitefly resistance of selected F**_**2**_ **BC**_**3**_ **plants and composition of the chromosomes 1, 5 and 12.** Different letters indicate statistical differences according LSD (α < 0.05). Areas filled in black represent homozygous markers (*S. habrochaites* CGN 1.1561 allele). Areas filled in grey represent heterozygous markers. Non-filled areas represent homozygous markers (cv. Moneymaker allele). On Chromosome 1 and 12 are indicated the physical region (tomato genome assemble version ITAG2.3) of *Tv-1* (76.7 to 90.0 Mbp) and *Tv-2* (4.6 to 63.5 Mbp).

Plant material development started from F_2_ plants containing *Tv-1*, *Tv-2* or both using the markers shown in Table 2. Three successive marker assisted backcrosses were carried out with selection for the presence of at least one of the markers linked to the QTL (Figure 1). Twenty-three F_2_BC_3_ plants were randomly selected for phenotyping and genotyping to confirm the presence of *Tv-1* and *Tv-2*. As reference lines, we included *S. habrochaites* (CGN1.1561) and *S. lycopersicum* cv. Moneymaker. Accession CGN1.1561 showed low values for adult survival (AS, 0.1 ± 0.21 females/day) and oviposition rate (OR, 0.2 ± 0.30 eggs/female/day), and cv. Moneymaker showed high values for adult survival (AS, 1.0 ± 0.01 females/day) and oviposition rate (OR, 5.5 ± 0.72 eggs/female/day). Among the twenty-three F_2_BC_3_, variation was found for both parameters. For AS, only three F_2_BC_3_ plants were significantly different from cv. Moneymaker (Figure 2). Whereas, for OR a gradient was observed, with fourteen F_2_BC_3_ plants showing statistically significant lower values than cv. Moneymaker (Figure 2). To determine the position and size of the introgressions, the twenty-three F_2_BC_3_ plants were genotyped using an Infinium bead array [40]. Several plants had an introgression of *Tv-1*, *Tv-2* or parts thereof. None of the plants had the complete *Tv-1* and *Tv-2* region as defined by Maliepaard *et al*. [28] (Figure 2). The four F_2_BC_3_ plants with the lowest OR (PV101092-2, PV101088-2, PV101087-3 and PV101088-5) shared an introgression on Chr. 5, but had differences in the presence of the regions *Tv-1* and/or *Tv-2*. One plant (PV101088-8) had the same introgression on Chr. 5, but the OR was not significantly different from cv. Moneymaker (Figure 2).

**Figure 3:**
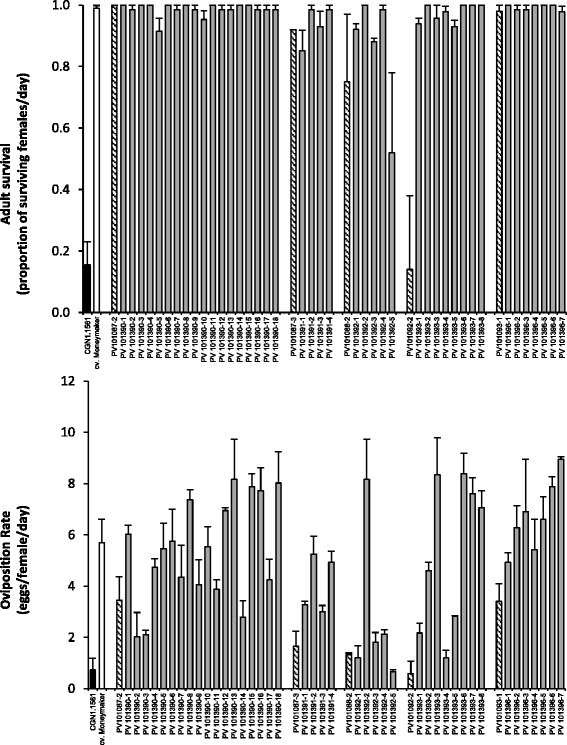
**Phenotyping results of the F**_**2**_ **BC**_**4**_ **plants.** The upper panel shows adult survival, the lower panel oviposition rate. Plants are grouped according to family. The first sample of each block is the parent of that family (black stripes). *Solanum habrochaites* (CGN1.1561) is black and cv. Moneymaker is white.

**Figure 4:**
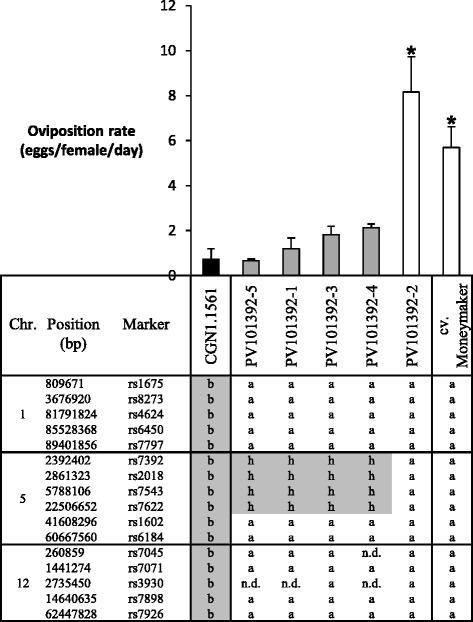
**Genotype and Phenotype of F**_**2**_ **BC**_**3**_ **PV101088-2 siblings.** Oviposition rate (mean ± standard error) and marker data for 5 F_2_BC_4_ siblings from the F_2_BC_3_ PV101088-2 line are given. Asterisks indicate statistical significance according LSD (P = 0.05). Marker score nomenclature: a = homozygous cv. Moneymaker allele; b = homozygous *S. habrochaites* (CGN 1.1561) allele; h = heterozygous; n.d. = no data. Chromosomal positions are according to International Tomato Annotation Group (ITAG) Release 2, official annotations on the SL2.31 can be found in [41], marker sequences in [40].

To further investigate the effect of the introgression on Chr. 5, five F_2_BC_3_ plants (PV101092-2, PV101088-2, PV101087-3, PV101093-1, PV101087-2) were selected based on OR, the presence/absence of *Tv-1*, *Tv-2* and the presence of the introgression on Chr. 5. The plants PV101092-2, PV101088-2, PV101087-3, contained the Chr. 5 (61.27 Mbp) introgression, whereas it was smaller in PV101087-2 and not present in PV101093-1. The five plants have varying parts of *Tv-1* and *Tv-2* or lack these completely (PV101088-2, Figure 2). The plants were backcrossed with cv. Moneymaker to generate five F_2_BC_4_ families. All F_2_BC_4_ plants plus parental plants, CGN1.1561 and cv. Moneymaker were genotyped for *Tv-1*, *Tv-2* and the introgression on Chr. 5, and phenotyped for adult survival, oviposition rate, trichome type and trichome density. Figure 3 shows the distribution for AS and OR and the link to the respective F_2_BC_3_ line. Clear differences were seen between cv. Moneymaker and CGN1.1561 for AS (P < 0.01) and OR (P < 0.01). In the studied F_2_BC_4_ plants, there was mainly segregation for OR with the parents on the extremes of the distribution. Genotyping showed that from the offspring of PV101088-2 (renamed to PV101392) four of the five sibling plants were heterozygous for the region on Chr. 5. These plants had an OR level comparable to CGN1.1561, the remaining plant of the five (PV101392-2), lacked the CGN1.1561 allele and had a high OR (Figure 4). To investigate a possible relation between the reduction in OR and the presence of glandular trichomes, the presence/density was determined on the parental lines and the F_2_BC_4_ plants. Accession CGN1.1561 was the only one with trichomes type IV and VIc, whereas the F_2_BC_4_ plants and the cv. Moneymaker had mainly trichomes type V and VIa. No differences were seen in the density of trichome type VIa among the F_2_BC_4_ plants and cv. Moneymaker.

**Figure 5:**
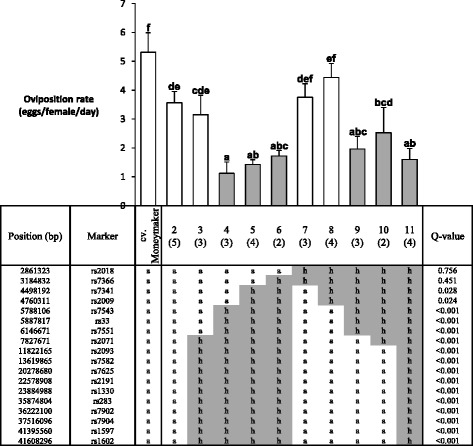
**Fine mapping of** ***OR-5*** **.** Oviposition rate (mean ± standard error) and marker data of F_2_BC_4_S_1_ plants grouped by introgressed fragment, based on marker scores. Different letters in oviposition rate graph indicate statistical significance according LSD (P = 0.05). The number of plants per specific introgression fragment is shown in brackets. Q-value: FDR corrected P-value per marker after t-test. Marker score nomenclature: a = homozygous cv. Moneymaker allele; b = homozygous *S. habrochaites* (CGN1.1561) allele; h = heterozygous. Chromosomal positions are according to International Tomato Annotation Group (ITAG) Release 2, official annotations on the SL2.31 can be found in [41], marker sequences in [40].

As the offspring of PV101392 showed a low OR and lacked the *Tv-1* and *Tv-2* region (Figure 4), we focussed on the introgression on Chr. 5. To find offspring plants with a smaller introgression on Chr. 5, PV101392-1PV101392-3, PV101392-4 and PV101392-5 were selfed. Of the 275 F_2_BC_4_S_1_ offspring plants, 33 plants out of 61 recombinants were selected based on length differences of the introgressed region, as judged from marker analysis. The genotyping results (grouped by introgression length) and phenotyping results (OR) are shown in Figure 5. With the F_2_BC_4_S_1_ we could narrow down this introgression to a 3.06 Mbp region between the markers rs2009 (4.76 Mbp) and rs2071 (7.83 Mbp).

**Figure 6:**
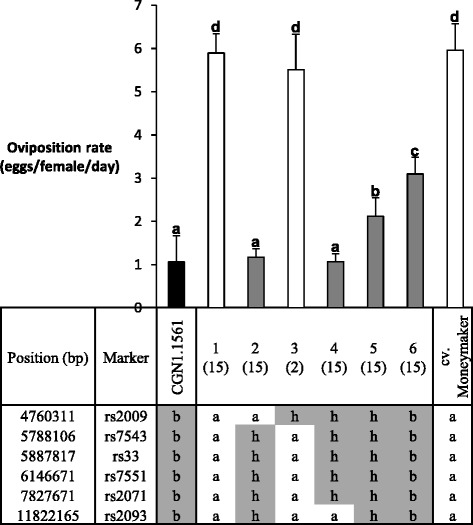
**Corroboration of role of** ***OR-5*** **.** Oviposition rate (mean ± standard error) and marker data of F_2_BC_4_S_2_ plants grouped by introgressed fragment, based on marker scores. Different letters in oviposition rate graph indicate statistical significance according LSD (P = 0.05). The number of plants per specific introgression fragment is shown in brackets. Marker score nomenclature: a = homozygous cv. Moneymaker allele; b = homozygous *S. habrochaites* (CGN 1.1561) allele; h = heterozygous. Chromosomal positions are according to International Tomato Annotation Group (ITAG) Release 2, official annotations on the SL2.31 can be found in [41], marker sequences in [40].

To further fine map and confirm the effect of the introgression on Chr. 5, eight F_2_BC_4_S_1_ plants (PV121430-4, PV121430-11, PV121433-30, PV121430-89, PV121432-26, PV121433-29, PV121433-53 and PV121434-57) with a low OR and heterozygous for parts of this region in Chr. 5 were selfed. Of the 295 F_2_BC_4_S_2_, 77 plants out of 154 recombinants were phenotyped based on length differences of the introgressed region, as judged from marker analysis. The results grouped by introgression length are shown in Figure 6. The F_2_BC_4_S_2_ plants with a *S. habrochaites* (CGN 1.1561) introgression on Chr. 5, between the markers rs2009 (4.76 Mbp) and rs2093 (11.8 Mbp), had an OR similar to the low levels of CGN1.1561 and in the case of plants with the cv. Moneymaker allele homozygous present, the OR was higher (Figure 6). Some of the F_2_BC_4_S_2_ had a reduced adult survival; however, AS and OR were not strongly correlated (*R* = 0.43) having plants with the introgression on Chr. 5 and with AS levels comparable to those found on cv. Moneymaker and with a significant lower OR. In the F_2_BC_4_S_2,_ no plants were found with a further smaller introgression.

Because CGN1.1561 is member of the group of *S. habrochaites* accessions that accumulate methyl ketones, the F_2_BC_4_S_2_ families were analysed for the presence of those compounds (2-Tridecanone, 2-Undecanone, 2-Pentadecanone and 2-Dodecanone). The accession CGN1.1561 had all these methyl ketones in higher relative abundance compared to cv. Moneymaker and to the F_2_BC_4_S_2_ families (Additional file 1: Table S1). In addition, there were no differences on the relative abundance of these methyl ketones among the F_2_BC_4_S_2_ families and cv. Moneymaker.

## Discussion

### An introgression on Chromosome 5 *(*OR-5*)* reduces whitefly oviposition rate

Using F_2_BC_3_ plants, we identified a *S. habrochaites* (CGN1.1561) introgression on the short arm of Chr. 5 (hereafter called *OR-5*), which confers a reduction in *B. tabaci* oviposition rate. By analysing F_2_BC_4,_ F_2_BC_4_S_1_ and F_2_BC_4_S_2_ populations, we could confirm that this introgression of 3.06 Mbp is causing the reduced whitefly oviposition rate. The reduction in oviposition caused by the presence of *OR-5* is independent of adult survival and the presence of trichome type IV. Plants were found on which all whiteflies were alive but a reduction in oviposition was observed (Figure 3) and none of the plants had the sticky trichomes type IV. The plants homozygous for the *S. habrochaites* (CGN1.1561) allele in the F_2_BC_4_S_2_ had a higher OR compared to plants heterozygous for this allele. This effect of over dominance might indicate an interaction between the *S. habrochaites* (CGN1.1561) and the *S. lycopersicum* allele. It would also implicate that the high level of resistance in terms of low AS and OR found in CGN1.1561 is the result of epistatic interaction between different genes. In the F_2_BC_3_ population only one plant had the *OR-5* region but with OR levels similar to cv. Moneymaker. This result may be explained in several ways. First, there is the chance of a double recombination in the *OR-5* region. However, no double recombination event was detected in this plant with the Infinium bead array. Secondly, there is the possibility of an epistatic effect between *OR-5* and a locus different from *Tv-1* or *Tv-2*. Finally, there is always the possibility of a phenotyping artefact.

### Selection of the chromosome 5 region

For the selection of the F_2_BC_3_ plants, we used markers that are linked to the loci *Tv-1* and *Tv-2* loci, which are located on Chr. 1 and 12 respectively. It is therefore remarkable that we ended up with an introgression on Chr. 5, which had never actively been selected for. This may be explained by starting with F_2_ plants containing the introgression on Chr. 5 either homozygous or heterozygous (3 out of 4 plants have the introgression). The chance that plants in the F_2_BC_3_ still possess the introgression is 1 out of 4 or 8, which is more or less the number of Chromosome 5 containing F_2_BC_3_ plants that we found. The fact that Maliepaard *et al.* [28] did not detect the QTL for OR could be caused by the different whitefly species used (*T. vaporariorum* vs. *B. tabaci*). Different insect species or biotypes may react differently to the same host plant or odour blend, resulting in different behaviour. For example, glucosinolates can confer resistance to some insects, whereas they can be used as host and strong oviposition cues for others [45]. In the case of whiteflies, differences were seen when compared the feeding behaviour of the Q and the B-biotype on the same host plant [46]. Also, tomato plants carrying the *Mi1-2* gene were in general more resistance to the Q-biotype than to the B-biotype [47].

### Nature of the resistance provided by OR-5

Several QTLs related to whitefly resistance have been identified on Chr. 5 (Table 1). Maliepaard *et al.* [28] found in the region of *OR-5* a QTL (*TriIV-1*) that increases the density of trichomes type IV. However, we did not detect any type IV trichomes on the F_2_BC_4_ plants containing the *OR-5* introgression. In a backcross population of potato ((*S. tuberosum* × *S. berthaultii*) × *S. berthaultii*) a region on Chr. 5 was associated with a reduction in the oviposition rate and leaf consumption by the Colorado potato beetle (*Leptinotarsa decemlineata*) [48]. This region also had a large effect on the density of the glandular secretory type B trichome (LOD: 19.17, explaining 35.6% of the variance), furthermore differences in the sucrose ester levels and in the presence of droplet (exudate) on the tip of the trichomes were associated with this region on Chr. 5 [48]. For *S. pennellii,* two QTLs were described on Chr. 5 that are involved in acyl sugar metabolism, one (*TA5*) related to the total accumulation of acyl sugars and another (*5*) related to the proportion of 7-methyloctanonate and 9-methyldecanoncate fatty acids that are incorporated into acyl sugars [49,50]. To check if acyl sugars were related to the reduction in oviposition rate an LC-MS chromatography analysis was done on the F_2_BC_4_S_2_ plants [14]. No differences were found among the F_2_BC_4_S_2_ plants on the levels of acyls sugars, pointing to a different mechanism of resistance in this plant material specifically affecting whitefly oviposition rate (data not shown). As the parental accession CGN1.1561 accumulates methyl ketones, we also analysed the offspring for the presence of 2-Tridecanone, 2-Undecanone, 2-Pentadecanone and 2-Dodecanone. None of these compounds was detected at elevated levels in the offspring, excluding the option that these methyl ketones may explain the observed reduction in oviposition rate. On the 3.06 Mbp introgression of *OR-5* are 258 annotated genes including R-genes, transcription factors, genes involved in acyl sugar and terpenoid metabolism which can be considered as candidate genes for reduced oviposition. To reduce the list of candidate genes and find the gene(s) responsible for the lower OR further fine mapping and functional analysis, including more detailed metabolomics is needed. However, considering the lack of recombinants found in the F_2_BC_4_S_1_ and F_2_BC_4_S_2_ populations between the markers rs-7543 (5.79 Mbp) and rs-7551 (6.15 Mbp), it might be difficult to reduce the size of the introgression.

### Perspectives of OR-5 for breeding whitefly resistant varieties

Since the late nineties of the 20th century, the efforts to get whitefly resistant tomatoes have increased considerably, but so far they have been unsuccessful [4]. The screening of genetic resources for novel whitefly resistance mechanisms has increased, going from distant wild relatives of tomato (i.e. *S. pennellii*, *S. habrochaites*) to in depth studies of several accessions of closely related species (i.e. *S. galapagense, S. pimpinellifolium*) [7-9,11,13,14]. These efforts have led to the identification of specific secondary metabolites conferring resistance to whiteflies (methyl ketones, sesquiterpenes, and acyl sugars) [12,15,51], the identification of QTLs related to resistance [16,28,33], and in some cases to the genes involved in the synthesis of resistance related metabolites [17,19,20,30,31]. The identification of *OR-5*, affecting specifically whitefly oviposition rate and independent of the presence of trichome type IV, opens new opportunities for breeding. The *OR-5* region is expected to reduce population development of *B. tabaci* strongly. As the reduction in oviposition is not linked to the sticky trichomes type I and IV, and the known negative effect of this type of resistance on parasitoids and predators [43,52-54], it can be expected that this resistance will be very suitable in combination with biological control. On varieties containing the *OR-5* region the *B. tabaci* population development will be slowed down giving the natural enemies ample opportunity to keep the population below threshold levels or even to remove developing whiteflies. Therefore, the gene will in particular be useful in protected tomato production conditions (greenhouse cultivation). For open field production, the resistances based on trichomes type I and IV will be more suitable [16].

## Conclusions

We identified a region at the top of Chr. 5 (OR-5), which is associated with a reduction in the oviposition rate of *B. tabaci*. This reduction was independent of the presence of the QTLs *Tv-1* and *Tv-2* that were identified previously [28], as well as of the presence of trichomes type IV. The OR-5 locus will provide new opportunities for resistance breeding against whiteflies, which is especially relevant in greenhouse cultivation.

## Additional file

Additional file 1: Table S1 GC-MS analysis of the relative abundance (average ± standard error) of methyl ketone per F_2_BC_4_S_2_ family.
