# Supplementary material for: Normal adult survival but reduced Bemisia tabaci oviposition rate on tomato lines carrying an introgression from S. habrochaites
Source: BMC Genet. 2014 Dec 24;15:142. doi: 10.1186/s12863-014-0142-3 (PMC4301655; doi:10.1186/s12863-014-0142-3)
Supplement: Additional file 1: Table S1 — GC-MS analysis of the relative abundance (average ± standard error) of methyl ketone per F2BC4S2 family. [file 12863_2014_142_MOESM1_ESM.docx]

Table S1: GC-MS analysis of the relative abundance (average ± standard error) of methyl ketone per F_2_BC_4_S_2_ family.

| **F_2_BC_4_S_2_ family (Recombinant class)** | **#** | **2-Tridecanone** | | **2-Undecanone** | | **2-Pentadecanone** | | **2-Dodecanone** | |
| --- | --- | --- | --- | --- | --- | --- | --- | --- | --- |
| 1 | (3) | 278 | ± 132 | 74 | ± 17 | 43 | ± 43 | 0 | ± 0 |
| 2 | (3) | 487 | ± 300 | 195 | ± 71 | 77 | ± 14 | 40 | ± 20 |
| 3 | (1) | 118 | - | 284 | - | 73 | - | 0 | - |
| 4 | (3) | 80 | ± 44 | 50 | ± 25 | 23 | ± 23 | 0 | ± 0 |
| 5 | (3) | 1608 | ± 921 | 275 | ± 79 | 408 | ± 309 | 40 | ± 40 |
| 6 | (3) | 117 | ± 20 | 31 | ± 31 | 27 | ± 27 | 34 | ± 17 |
| cv. Moneymaker | (1) | 692 | - | 65 | - | 96 | - | 0 | - |
| CGN1.1561 | (1) | 3171786 | - | 664846 | - | 30897 | - | 4347 | - |

Per F_2_BC_4_S_2_ family tree biological replicas consisting of 5 pooled plants each were used. Due to the low number of recombinants obtained for the family number 3, only one replica consisted on 2 pooled plants was used. For the references lines (cv. Moneymaker and CGN1.1561) one replica was analysed consisting of 5 pooled plants.
